# T cell receptor transgenic lymphocytes infiltrating murine tumors are not induced to express foxp3

**DOI:** 10.1186/1756-8722-4-48

**Published:** 2011-11-23

**Authors:** Jon G Quatromoni, Lilah F Morris, Timothy R Donahue, Yue Wang, William McBride, Talal Chatila, James S Economou

**Affiliations:** 1 Departments of Surgery, University of California, Los Angeles, 10833 Le Conte Avenue Room 54-140 CHS Los Angeles, CA 90095 USA; 2 Radiation Oncology, University of California, 10833 Le Conte Avenue, Room B3-109 CHS Los Angeles, CA 90095 USA; 3 Pediatrics, University of California, 805 Tiverton, Room 12-430 Marion Davies Children's Center, Los Angeles, CA 90095 USA; 4 Microbiology, Immunology and Molecular Genetics+, University of California, Los Angeles, 10833 Le Conte Avenue, Room 54-140 CHS, Los Angeles, CA 90095 USA; 5 Molecular and Medical Pharmacology, University of California, 10833 Le Conte Ave, 54-140 CHS, Los Angeles, CA 90095 USA

## Abstract

Regulatory T cells (Treg) that express the transcription factor Foxp3 are enriched within a broad range of murine and human solid tumors. The ontogeny of these Foxp3 Tregs - selective accumulation or proliferation of natural thymus-derived Treg (nTreg) or induced Treg (iTreg) converted in the periphery from naïve T cells - is not known. We used several strains of mice in which Foxp3 and EGFP are coordinately expressed to address this issue. We confirmed that Foxp3-positive CD4 T cells are enriched among tumor-infiltrating lymphocytes (TIL) and splenocytes (SPL) in B16 murine melanoma-bearing C57BL/6 Foxp3^EGFP ^mice. OT-II Foxp3^EGFP ^mice are essentially devoid of nTreg, having transgenic CD4 T cells that recognize a class II-restricted epitope derived from ovalbumin; Foxp3 expression could not be detected in TIL or SPL in these mice when implanted with ovalbumin-transfected B16 tumor (B16-OVA). Likewise, TIL isolated from B16 tumors implanted in Pmel-1 Foxp3^EGFP ^mice, whose CD8 T cells recognize a class I-restricted gp100 epitope, were not induced to express Foxp3. All of these T cell populations - wild-type CD4, pmel CD8 and OTII CD4 - could be induced in vitro to express Foxp3 by engagement of their T cell receptor (TCR) and exposure to transforming growth factor β (TGFβ). B16 melanoma produces TGFβ and both pmel CD8 and OTII CD4 express TCR that should be engaged within B16 and B16-OVA respectively. Thus, CD8 and CD4 transgenic T cells in these animal models failed to undergo peripheral induction of Foxp3 in a tumor microenvironment.

## Background

Treg play an essential role in maintaining immunological self-tolerance [1]. Approximately 10% of CD4 T cells express the transcription factor FoxP3 (forhead box P3 transcription factor); humans and mice with inactivating Foxp3 mutations have autoimmune diseases [2-4]. Treg dominantly suppress immune responses through direct contact with dendritic cells, effector T cells and possibly through secretion of immunosuppressive cytokines [5,6]. Fewer than 1% of CD8 T cells express Foxp3, and the biology of this very small population of naturally occurring, thymus-derived T cell have not been well studied. However, this transcription factor can be induced in both CD4 and CD8 T cells through engagement of their T cell receptors (TCR) and exposure to transforming growth factor beta (TGFβ) [7-10]. These so called "induced" Treg (iTreg), both CD4 and CD8, can acquire dominant suppressor phenotype in a variety of experimental models [11-13].

Many studies have shown that the number of Treg are significantly increased in the peripheral blood, bone marrow, tumor draining lymph nodes, and TIL of mice and humans bearing many types of hematologic and solid malignancies including breast [14], colorectal [15], esophageal [16], gastric [17], hepatocellular [18], lung [19], melanoma [20], ovarian [21], and pancreatic cancers [14]. It has been hypothesized that these Treg may be involved with promoting tumor progression, as they are even more enriched in advanced tumors [22]. The number of Foxp3 Treg within human tumors has also been correlated with a poorer prognosis. Patients with ovarian or gastric cancer and lower numbers of Treg TILs had improved disease-specific survival [23]; those with head and neck cancer also experienced better locoregional control [24]. Treg isolated from human ovarian cancers were able to inhibit Her-2 specific CD8+ effector responses, as measured by proliferation, cytotoxicity, and IL2 and IFNγ production [25]. These and other observations support the view that Foxp3 Treg may dominantly suppress antitumor immune responses. The ontogeny of the enriched Treg population found within tumors, generally CD4, is not fully defined. A selective tumor-driven accumulation or proliferation of thymus-derived natural (n)Treg is a possibility. Alternatively, naïve Foxp3 T cells could be induced to express this regulatory transcription factor through tumor-derived signals yielding induced (i)Treg. These signals would include engagement of TCR and exposure to TGFβ elaborated by tumors or tumor-associated stroma. We sought to address this question by generating CD8 (Pmel-1) and CD4 (OTII) TCR transgenic mice in which Foxp3 expression could be detected by EGFP expression (Foxp3^EGFP^). These naïve Pmel-1 CD8 and OTII CD4 populations have very low to absent Foxp3 expression but could be induced in T cells in vitro with a combination of T cell receptor (TCR) engagement and TGFβ signaling. We reasoned that both of these TCR transgenic cell populations, entering B16 or ovalbumin-transfected B16 (B16-OVA) subcutaneous tumors respectively, would be exposed to a comparable set of Foxp3 induction signals. CD4/Foxp3^EGFP ^cells are enriched in B16 tumors and spleen when tumors are propagated in wild-type C57BL/6 Foxp3^EGFP ^mice. However, in neither TCR transgenic mouse did we find evidence of Foxp3 induction among tumor-infiltrating lymphocytes (TIL), splenocytes (SPL) nor lymph nodes (LN). These findings argue indirectly in favor of a preferential accumulation of nTreg in experimental tumors.

## Materials and methods

### Mice

Mice were bred and kept under defined-flora pathogen-free conditions at the American Association of Laboratory Animal Care-approved Animal Facility of the Division of Experimental Radiation Oncology, University of California, Los Angeles. Mice were handled in accordance with the University of California animal care policy. Foxp3^EGFP ^mice were derived as previously described [26] and backcrossed for 12 generations on the C57BL/6 background. The following mouse strains, all on C57BL/6 background, were obtained from the Jackson Laboratory: Recombinase activating gene 1 (RAG1)-deficient mice, Pmel-1 TCR transgenic mice that recognize the MHC class I (H-2 D^b^)-restricted epitope of gp100 (25-33) presented on the surface of B16 melanoma, and OTII TCR transgenic mice whose transgenic receptor recognizes ovalbumin 323-339 in the context of I-A^b^. Pmel-Foxp3^EGFP ^mice, and OTII-RAG1KO-Foxp3^EGFP ^mice (OTII-Foxp3^EGFP ^mice) were derived by crossing the respective mice.

### Tumors cell lines and adoptive therapy

The murine melanoma cell line B16 was obtained from the American Type Culture Collection and maintained in Dulbecco's modified Eagle's medium plus 10% FBS, penicillin (100 U/ml), streptomycin (100 μg/ml), Amphotericin B (0.25 μg/ml). B16-OVA (expressing the gene for ovalbumin peptide), a kind gift from Protul Shrikant, was cultured in RPMI (Mediatech Cell Gro) 1640 medium as described above, plus G418 (Invitrogen, 400 μg/ml). IL-2 was a kind gift from Chiron Corporation (Novartis).

### Adoptive cell therapy

Adoptive cell therapy, mouse irradiation, bone marrow transplantation and systemic IL-2 administration were conducted as previously described [27]. Specific details with respect to cell numbers may be found in the footnote to Table 1.

**Table 1:** Spleen cells were isolated and cultured for 72 hours under the indicated conditions with the following concentrations: IL-2 (100 u/ml), TGFβ (10 ng/ml) and αCD3/αCD28 (1 μg/ml immobilized, 10 μg/ml soluble, respectively).

Culture Conditions	C57BL/6 Foxp3^EGFP^ CD8	C57BL/6 Foxp3^EGFP^ CD4	Pmel Foxp3^EGFP^ CD8	Pmel Foxp3^EGFP^ CD4	OTII Foxp3EGFP CD4
Media Only	0.9 +/- 0.6	2.1 +/- 1.3	2.2 +/- 0.1	9.6 +/- 5.9	0.8 +/- 0.1
IL-2	1.9 +/- 0.5	8.2 +/- 1.8	2.9 +/- 0.1	11.7 +/- 1.3	0.6 +/- 0.3
TGF- β	1.8 +/- 0.1	4.2 +/- 1.1	4.8 +/- 0.8	13.2 +/- 1.6	0 +/- 0.6
IL-2/TGF-β	2.0 +/- 1.3	20.4 +/- 1.4	1.4 +/- 0.8	20.7 +/- 3.4	0.8 +/- 0.5
αCD3/αCD28	0.9 +/- 1.8	3.1 +/- 0.2	2.3 +/- 0.3	17.2 +/- 1.7	3.0 +/- 0.1
αCD3/αCD28/IL-2	1.3 +/- 1.6	4.6 +/- 0.7	21.3 +/- 0.8	13.8 +/- 0.5	3.0 +/- 0.2
αCD3/αCD28/TGF-β	18.8 +/- 3.0	18.8 +/- 1.0	27.3 +/- 1.8	30.7 +/- 2.8	7.7 +/- 2.4
αCD3/αCD28/IL-2/TGFβ	25.1 +/- 0.4	34.4 +/- 1.1	27.9 +/- 0.2	46.2 +/- 1.1	8.4 +/- 0.4

Cells were then sorted for CD8, CD4, and EGFP expression. Results are a mean of triplicates +/- S.D. These experiments were repeated at least 3 times with comparable results.

### Flow cytometry

A single cell suspension was prepared from spleens and lymph nodes in PBS by filtering through a 0.7 μm mesh cell strainer. A total of 10^6 ^splenocytes were then labeled with mAb mixtures (BD Pharmingen) to CD3^PerCP^(5 μg/ml), CD8α^PE ^(5 μg/ml), and/or CD4^APC ^(5 μg/ml). Non-specific antibody binding was blocked on cells with Fc-blocking solution (5 μg/ml) for 10 minutes. Cells were then labeled for 30 min on ice in the dark, washed, fixed (if analysis was to take place on following day), and analyzed. Stained cells were collected and analyzed on a FACSCalibur machine, using CellQuest software, and numbers of T cells populations and % EGFP-positive cells were measured. To isolate tumor-inflitrating lymphocytes (TIL), a single cell suspension was prepared in the following manner: tumors were minced with sterile surgical blades in 5 mL PBS in a petri dish, washed twice with PBS, enzymatically digested in 0.1% Dispase II solution (Roche) for 40 min at room temperature with gentle mixing, and re-suspended in PBS. RBC were lysed with 1× PharmLyse (BD Pharmingen), and cells were washed, re-suspended in RPMI 1640 medium with 10% FBS and 1% PSF, and counted. The numbers of TIL isolated varied widely and correlated to a large degree with the size of the tumor. As few as several thousand to greater than a half million TIL could be isolated from each tumor. The entire lymphocyte population from each tumor was analyzed by flow cytometry. Additional files 1 and 2 show gating strategies for Foxp3^EGFP ^T reg isolated from mice or generated in vitro, respectively.

### TGFβ ELISA assay

Freshly explanted B16 tumor cells, B16 cultured cells, or B16-OVA cultured cells were plated in one well of a six-well plate in described media on day 0. On day 2, cells were washed and resuspended in 2 mL serum-free and antibiotic-free DMEM or RPMI. On day 3, when the cells were 100% confluent, supernatants were collected. Supernatant TGFβ concentrations were measured using ELISA (Mouse/Rat/Porcine/Canine TGFβ 1 Quantikine ELISA Kit; R&D Systems) according to the manufacturer's recommendation.

### Statistical analysis

Statistical analysis and P-value determinations were done by two-tailed Welch's T-test for determination of the significance of differences between the groups of continuous variables. P < 0.05 was considered to be statistically significant. Graphs were constructed using GraphPad Prism 4.0 software and statistical functions were analyzed using Microsoft Excel.

## Results

### Foxp3-positive CD4 T cells are enriched in tumor-infiltrating lymphocytes

Many groups have reported high percentages of Foxp3-positive lymphocytes infiltrating both murine and human tumors and have postulated that these Treg may dominantly suppress an antitumor immune response [25,28]. We confirmed this in C57BL/6 Foxp3^EGFP ^mice in which Foxp3/Treg are identified by EGFP expression; in these mice an IRES-linked EGFP is bicistronically co-expressed with Foxp3 under control of the endogenous Foxp3 promoter/enhancer.[29] B16 melanoma was propagated subcutaneously in these mice. At various tumor volumes, tumor-infiltrating lymphocytes (TIL), splenocytes (SPL), and lymph node (LN) cells were isolated and CD8 and CD4 T cells analyzed by flow cytometry for percentage expression of Foxp3 (EGFP). A significantly higher percentage of CD4 TIL expressed Foxp3 compared with CD4 SPL (30% vs. 13%, p = 0.016, Welch's T-test for unequal variances). Treg were enriched in spleens but not lymph nodes of tumor-bearing animals compared with healthy controls (p = 0.003). A very low percentage (<1%) of CD8 T cells express Foxp3; this was unchanged in tumor-bearing mice.

#### T cell receptor transgenic CD8 and CD4 T cells are not induced to express Foxp3 in tumor-bearing mice

The ontogeny of these enriched Foxp3-positive cells in tumor-bearing hosts is still a matter of controversy: do they represent thymus-derived natural Treg (nTreg) or induced Treg (iTreg) derived from naïve T cells in which Foxp3 expression is induced through some tumor-dependent mechanism? To address this question, we crossed C57BL/6 Foxp3^EGFP ^mice (on a RAG-1 -/- background) with Pmel mice (on a wild-type background) and with OTII mice (on a RAG-1 -/- background). Mice yielded from the former cross (Pmel/Foxp3^EGFP^) express the pmel TCR on approximately 70% of their CD8 T cells. This TCR recognizes the MHC class I (H-2D^b^)-restricted epitope of gp100 (25-33) presented on the surface of B16 melanoma; adoptively transferred pmel CD8 T cells can mediate regression of B16 in vivo. Generally fewer than 0.3% of Pmel CD8 express Foxp3^EGFP^. The biology CD4 T cell population in these Pmel mice have not been studied; we noted an increased percent expression of Foxp3 in CD4s from both control and tumor-bearing mice, and to a comparable degree. Other investigators (R. Prins) have reported that CD4s from these mice also express the Pmel TCR, but we did not investigate this further as this study was focused on CD8 Pmel population. Mice yielded from the latter cross (OTII/Foxp3^EGFP^) are devoid of CD8s and express a TCR on their CD4 T cells that recognize a class II (H-2IA^6^)-restricted epitope (323-339) derived from chicken ovalbumin; these mice lack nTreg because OVA is not presented in the thymus.

We propagated B16 and B16-OVA tumors in Pmel Foxp3^EGFP ^and OTII Foxp3^EGFP ^mice, respectively. At various tumor volumes, SPL, LN, and TIL were analyzed for percent expression of Foxp3^EGFP ^in CD8 and CD4 T cells. In Pmel/Foxp3^EGFP ^mice, we did not observe any significant enrichment of CD8/Foxp3^EGFP ^positive cells in these sites. Higher percentages of CD4/Foxp3 positive cells were found in spleens and LN from nontumor-bearing Pmel/Foxp3^EGFP ^mice compared to C57BL/6 Foxp3^EGFP ^mice (overall average 25% compared with 12%), but there was no additional Foxp3 enrichment among CD4 TIL in tumor-bearing animals. In OTII/Foxp3^EGFP ^mice, at most 0.2% of CD4 T cells retrieved from B16-OVA tumor-bearing mice express Foxp3^EGFP^.

### Signaling requirements for Foxp3 induction in TCR transgenic CD8 and CD4 T cells

These in vivo studies suggested that naïve Foxp3-negative CD8 and CD4 T cells could not be induced to express Foxp3 after they have infiltrated and been exposed to signals within the tumor microenvironment. We confirmed that naïve Foxp3-negative CD4 and CD8 populations, both TCR-transgenic and nontransgenic, were capable of being induced to express Foxp3^EGFP ^by in vitro TCR engagement (αCD3 and αCD28) and exposure to TGFβ (Table 1). Cells cultured from freshly explanted B16 tumors produced TGFβ at levels ranging from 3.5 to 4.0 ng/mL, three-fold higher than that produced by B16 cells maintained in culture (data not shown), possibly because of contamination by tumor-associated macrophages. This nanogram concentration level of TGFβ in the tumor microenvironment should be adequate to induce Foxp3.

**Figure 1:**
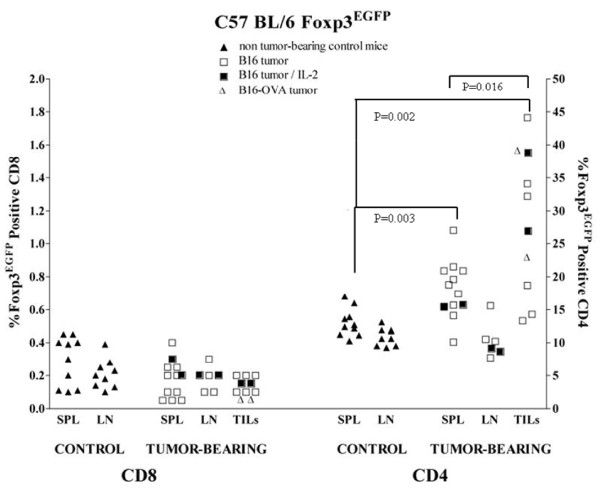
**Expression of Foxp3^EGFP ^in T cells from tumor-bearing and control mice**. Splenocytes (SPL), lymph node lymphocytes (LN), and tumor-infiltrating lymphocytes (TIL) were isolated from control and B16 tumor-bearing C57BL/6 Foxp3 ^EGFP ^mice and analyzed by flow cytometry for Foxp3^EGFP ^expression in CD8 and CD4 lymphocytes. B16 (boxes) or B16-OVA (open triangles) tumors were allowed to grow to 7-10 mm in diameter at which point they either did (closed boxes) or did not receive daily intraperitoneal injections of 50,000 units IL-2 for three days prior to analysis on the following day. Foxp3^EGFP ^expression in CD8 T cells from control and tumor-bearing mice were at equivalently low percentages. The percent of CD4 cells that expressed Foxp3^EGFP ^in SPL (p = 0.002) and TIL (p = 0.001) was significantly elevated in tumor-bearing mice compared with non-tumor-bearing controls. Statistical analysis and P-value determinations were done by two-tailed Welch's T-test for determination of the significance of differences between the groups of continuous variables. P < 0.05 was considered to be statistically significant. Graphs were constructed using GraphPad Prism 4.0 software and statistical functions were analyzed using Micorsoft Excel.

**Figure 2:**
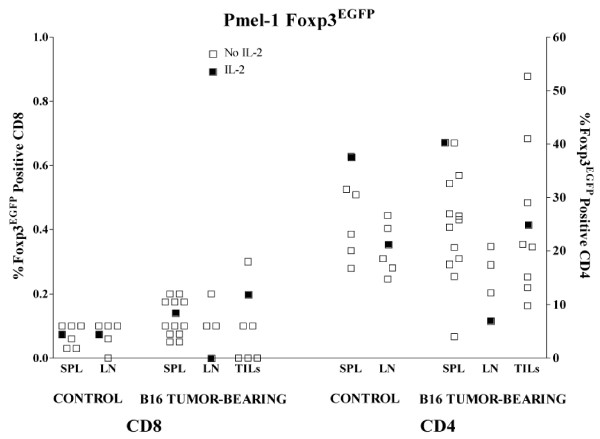
**Expression of Foxp3^EGFP ^in Pmel-1 transgenic mice**. The relative percentages of Foxp3^EGFP ^expression were comparable in splenocytes (SPL), lymph node lymphocytes (LN), and tumor-infiltrating lymphocytes (TILs) from control or B16-tumor-bearing Pmel Foxp3^EGFP ^mice. B16 tumors were allowed to grow to 7-10 mm in diameter at which point they either did (closed boxes) or did not (open boxes) receive daily intraperitoneal injections of 250,000 units IL-2 for three days prior to analysis on the following day.

**Figure 3:**
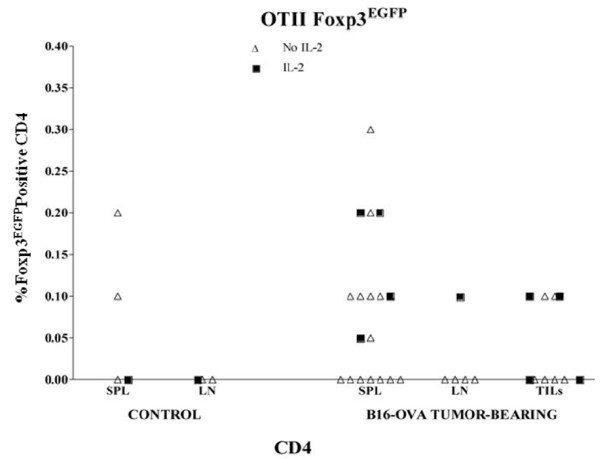
**Expression of Foxp3^EGFP ^in OTII transgenic mice**. The relative percentages of Foxp3^EGFP ^expression were comparable in splenocytes (SPL), lymph node lymphocytes (LN), and tumor-infiltrating lymphocytes (TIL) from control or B16-OVA-tumor-bearing OTII Foxp3^EGFP ^mice. B16-OVA tumors were allowed to grow to 7-10 mm in diameter at which point they either did (closed square) or did not (open triangle) receive daily intraperitoneal injections of IL-2 for three days prior to analysis on the following day. In this cohort, mice did not all receive the same dose of IL2: the non-tumor bearing mouse received 250,000 units IL-2 per injection while mice in the tumor-bearing group received either 100 (one mouse), 250 (three mice), or 500 (one mouse) thousand units per injection.

IL-2 plays an important role in Treg maintenance and iTreg development (ref). To overcome any deficiency in this regard, we administered systemic IL-2 (50-250 × 10^5 ^IU/mouse/day ×3 days prior to sacrifice) to several tumor-bearing animals of all three strains (shown as closed squares in Figures 1, 2 &3) but the proportions of Foxp3^EGFP ^cells in these mice were unchanged.

#### Adoptive transfer of Foxp3-negative CD4 and CD8 T cells into tumor-bearing mice

We finally asked if Foxp3-negative CD4 or CD8 T cells, adoptively transferred into tumor-bearing mice, could be induced to express this transcription factor. Populations of Foxp3^EGFP^-positive and-negative CD4 and CD8 T cells were adoptively transferred to C57BL/6 wild type B16-OVA-bearing mice. Adoptively transferred cell populations included: C57BL/6 CD4, nTreg, and iTreg; OTII CD4 and iTreg; and Pmel CD8s (see footnote to Table 2 for details). Prior to adoptive transfer, mice underwent a myeloablative regimen (900cGy) to allow repopulation of lymphoid organs by the administered T cells. Mice were supported with bone marrow from C57BL/6 on a CD45.1 background to allow unequivocal identification of adoptively transferred CD45.2 cells. All mice received systemic IL-2 (50,000 units intraperitoneal) on days 1, 2, and 3 after adoptive transfer. At intervals after adoptive therapy, splenocytes were isolated from recipient mice and analyzed for CD45.2, CD8 or CD4, and EGFP expression.

**Table 2:** Adoptive transfer of CD4 T cells populations into B16-OVA tumor-bearing mice.

Percent Foxp3^EGFP ^Positive Cells Received From Spleen and Tumor						
T Cell Subsets Adoptively Transferred	Spleen day 5	Tumor day 5	Spleen day 10	Tumor day 10	Spleen day 15	Tumor day 15
Bone marrow control (0%)	0.0, 0.0		0.0, 0.0		0.0, 0.0	
C57BL/6 CD4 (0%)	0.0, 0.0		0.3, 0.5	0.0,0.0	0.0, 0.0	
C57BL/6 nTreg CD4 (100%)	1.4, 1.9		11.7, 15.2		14.8, 20.8	
C57BL/6 iTreg CD4 (26%)	0.4, 0.3		4.5, 3.8		0.4, 2.0	
OTII CD4 (0%)	0.0, 0.1		0.0, 0.0		0.0, 0.0	0.0, 0.0
OTII iTreg CD4 (10.4%)	0.2, 0.0	0.0, 0.0	0.9, 1.4	0.1, 0.1	0.3, 0.4	
	**SPLEEN** **day 7**	**TUMOR** **day 7**	**SPLEEN** **day 14**	**TUMOR** **day 14**	**SPLEEN** **day 25**	**TUMOR** **day 25**
Pmel CD8 (<1%)	0.0, 0.0	0.1, 0.1	0.0, 0.0	0.1, 0.1	0.0, 0.0	0.0,0.0

C57BL/6 (CD45.2) mice bearing (five-day) B16-OVA tumors were irradiated with 900 cGy. The following day, all mice received bone marrow, from syngenic CD45.1 mice. Several different populations of cells were adoptively transferred into B16-OVA tumor-bearing mice. These cells were activated for 72 hours with αCD3/αCD28/IL-2, and in some cases flow-purified to yield a homogenous population of Foxp3^EGFP^-positive or negative cells. These cell populations included: (1) 10^5 ^C57BL/6 CD4 T cells (0% Foxp3^EGFP^); (2) 10^5 ^C57BL/6 CD4 nTreg (100% Foxp3^EGFP^); (3) 10^5 ^C57BL/6 CD4 TGFβ-induced iTreg (flow purified Foxp3^EGFP^-negative CD4 T cells, activated in the presence of TGFβ for 72 hours to yield ~26% Foxp3^EGFP ^iTreg); (4) 10^5 ^OTII CD4 T cells (0% Foxp3^EGFP^); (5) 10^5 ^OTII CD4 iTreg (10.4% Foxp3^EGFP^); and 10^6 ^Pmel CD8 (<1% Foxp3^EGFP^). When sufficient numbers of TIL could be isolated for flow (at least 10^3^), these were also surface phenotyped.

Shown in Table 2 is the percent Foxp3^EGFP ^expression for administered CD4 and CD8 T cells retrieved from spleen and TIL. Administered nTreg and iTreg could be recovered from the spleens of recipient mice. However, we did not detect induction of Foxp3 in repopulated spleens of any of the other administered populations of Foxp3^EGFP^-negative CD4 or CD8 T cells. Insufficient numbers of TIL were recovered in most groups to provide a reliable analysis.

## Discussion

We hypothesized that naïve T cells entering the tumor microenvironment would be exposed to the signals and factors needed for induction of Foxp3 and the acquisition of a regulatory phenotype. Some of these signals have been defined in vitro and include TCR engagement and exposure to TGFβ. We used TCR transgenic mice - one devoid of CD4 nTreg and the other with < 1% Foxp3 CD8s - whose tumor-infiltrating T cells and perhaps those in lymphoid organs of tumor-bearing mice would be engaged by cognate antigen, as well as being exposed to tumor-produced TGFβ. We demonstrated that naïve Foxp3-negative CD4 and CD8 populations, both transgenic and nontransgenic, could be induced to express Foxp3 in vitro by αCD3/αCD28 and TGFβ treatment (see Table 1). These in vitro findings confirm that Foxp3 expression can be induced in both T cells subtypes, and to comparable degrees, using similar sets of signals. We confirmed a significant enrichment of Foxp3-positive CD4 TIL and splenocytes in wild-type, B16 tumor-bearing C57BL/6 Foxp3^EGFP ^mice. However, neither CD8s in these mice, nor TCR transgenic T cells in either Foxp3^EGFP ^cross, seemed to have encountered the necessary intratumoral signals to induce Foxp3 expression. Administration of systemic IL-2, which we have shown in vitro to act in concert, but not alone, with TGFβ to induce Foxp3, did not support induction of iTreg in these transgenic mice. These findings indirectly support the view that natural, thymus-derived Treg preferentially accumulate, or proliferate, in the tumor microenvironment.

Two subsets of Tregs are recognized - adaptive or induced (iTreg) and natural (nTreg) - which together are responsible for maintaining tolerance to self-antigen and preventing autoimmune diseases or inappropriate immune responses involved in allergic diseases through the suppression of auto-reactive T cells [30,31]. Both require TGF-β and IL-2 for maintenance; express similar phenotypic markers such as CTLA4, GITR, CD25, and CCR4; and require the expression of Foxp3 to carry out a contact-dependent mechanism of action [32]. Where these two developmentally distinct populations differ is in their antigen specificities, strength of TCR stimulation and co-stimulatory signals required for their generation, and their stability of suppressive action. nTreg are generated in the thymus in a CD28-dependent manner, constitutively express CD25, express TCRs specific for self-antigen, demonstrate a more stable expression of Foxp3, and exert suppressive function [30-32]. Conversely, iTregs are generated in the peripheral lymphoid organs through the de novo conversion of CD4^+^CD25^-^Foxp3^- ^T cells in a TGF-β- and IL-2-dependent-manner, have TCRs specific for foreign antigens presented by professional antigen-presenting cells, require weaker TCR stimulation (CD28 stimulation not required), and demonstrate a less stable expression of Foxp3 [30-34].

Possible mechanisms for Treg enrichment within tumors include: preferential accumulation of nTreg, proliferation of nTreg within tumors, or peripheral conversion of naïve T cells to iTreg. Each mechanism is dependent upon tumor-derived signals (chemokines, cytokines, TCR engagement). One hypothesis is that resident nTreg proliferate in the tumor microenvironment [35]; another is that nTregs may weakly perceive tumor related signals and be selectively recruited where they may exert their suppressive function [25]. Treg can migrate more efficiently into major non-lymphoid tissue sites, such as tumors, due to their up-regulation of non-lymphoid tissue-specific homing receptors [36]. In human ovarian carcinoma, tumor cells and tumor-associated macrophages were shown to produce a Treg-specific chemokine CCL22, which resulted in the specific recruitment of Treg to the tumor microenvironment via their CCR4 receptor [25]. Interestingly, the CCR4 receptor is expressed at higher levels in Treg than in effector T cells in leukemia studies, suggesting its up-regulation may be Treg-specific [37]. In this same ovarian cancer model, IL-2 treatment was shown to up-regulate CCR4 as well as CXCR4, which further enhanced the ability of Treg to migrate to the tumor microenvironment based on its elevated levels of the Treg ligands CCL22 and CXCL12 [38,39]. Similar results were seen in a gastric cancer model, in which elevated levels of CCL17 and CCL26 in the tumor microenvironment demonstrated a positive correlation with the frequency of Foxp3 Treg, which can bind both these ligands with its CCR4 receptor [40]. Furthermore, Treg had a higher affinity for CCL17 and CCL22 than effector T cells in vitro as determined by a migration assay.

Expression of the regulatory cytokine TGFβ is abundant in many tumors, particularly in advanced stages. TGFβ can promote proliferation of Treg in vivo; the addition of IL-2 to TGFβ can induce Treg both in vivo and in vitro [41]. A number of additional chemokines and cytokines are implicated in Treg proliferation or induction, including IFNγ, IL-6, IL-23, IL-21, Cox2, and indolamine 2,3 dioxygenase [22,23,42-45]. Convincing in vivo evidence that iTreg develop from conventional CD4^+^CD25^- ^T cells was shown a non-obese diabetic (NOD) model, which normally exhibits extensive autoimmune manifestations around 12-16 weeks of age. When CD28^-/- ^NOD mice were treated with αCD3, which has been shown to increase CD4^+^CD25^+ ^T cells and result in long-term remission of disease in regular NOD, CD4^+^CD25^+ ^T cells were generated de novo and shown to be suppressive in vitro [46]. Neutralizing anti-TGF-β given to these CD28^-/- ^NOD mice alongside αCD3 prevented the aforementioned disease remission, demonstrating the role of TGF-β in the generation of iTreg and further supporting previous in vitro results.

Linehan [47,48] demonstrated that naïve CD4 T cells were converted into Foxp3-positive Treg when administered to Rag1-/- mice bearing TGFβ-producing (Pan 02) tumors. This conversion could be inhibited by TGFβ neutralizing antibody, was abrogated if naïve T cells were obtained from mice whose T cells were insensitive to TGFβ signaling, and did not occur in mice bearing tumors that did not produce TGFβ (ESO 2). The high levels of TGFβ expression by this murine pancreatic tumor cell line, and the longer interval (seven weeks) of in vivo co-residence of naïve CD4 T cells and tumor, may explain their clearcut but different findings.

## Conclusion

In summary, we generated two TCR transgenic mouse strains in which the de novo generation of iTreg could be unequivocally demonstrated in vitro, and in which putative signals for Foxp3 induction were present in the tumor microenvironment. Implantation of the B16 melanoma in wild-type mice resulted in the enrichment of CD4 Foxp3 Treg in TIL and spleen; this tumor did not induce an enrichment of either CD4 or CD8 Foxp3 in these two transgenic mouse strains.

## Abbreviations

(Cox2): Cyclooxygenase-2; (EGFP): Enhanced Green Fluorescent Protein; (IFNγ): interferon; (IL-2): Interleukin; (IL-6): Interleukin; (IL-21): Interleukin; (IL-23): Interleukin; (IU): International Units; (LN): Lymph Node; (NOD): Non-Obese Diabetic; (OVA): Ovalbumin; (SPL): Splenocytes; (TCR): T Cell Receptor; (Treg): Regulatory T cells; (iTreg): Induced Regulatory T cells; (TIL): Tumor Infiltrating Lymphocytes; (TGFβ): Transforming Growth Factor Beta.

## Competing interests

The authors declare that they have no competing interests.

## Authors' contributions

The experiments were carried out by JQ, LM, TD, YW. Mice were bred by TC. JE, TC, and WM conceived of the study. All the authors participated in the design and study coordination. The manuscript was drafted by JQ, LM and JE. All the authors read, edited and approved the final manuscript.

## Authors' information

JQ is a medical student. LM and TD are surgery residents. YW is a postdoctoral fellow. WM, TC and JE are faculty.

## Supplementary Material

Additional file 1 **Gating Strategies for Treg populations isolated from mice**. This file shows representative examples of gating for CD8 and CD4 Foxp3^EGFP ^cells from the spleens and tumors from different transgenic mice. These mice have been well described in previous publications where gating dot plates have been illustrated (see ref #26 which describes the generation of these mice by our coauthor T. Chatila). The numbers of infiltrating transgenic T cells varies with the size of the tumor and time after infusion. See figures 1, 2, 3, Table 2. Click here for file

Additional file 2 **Gating Strategies for Treg populations generated in vitro**. This file shows representative examples of gating for CD8 and CD4 Foxp3^EGFP ^cells generated in culture by different activated cultures. See Table 1. Click here for file
