# Supplementary material for: T cell receptor transgenic lymphocytes infiltrating murine tumors are not induced to express foxp3
Source: J Hematol Oncol. 2011 Nov 23;4:48. doi: 10.1186/1756-8722-4-48 (PMC3245424; doi:10.1186/1756-8722-4-48)

BL/6-Foxp3^{eGFP} CD4 and CD8 Populations In B16 Tumor (3)

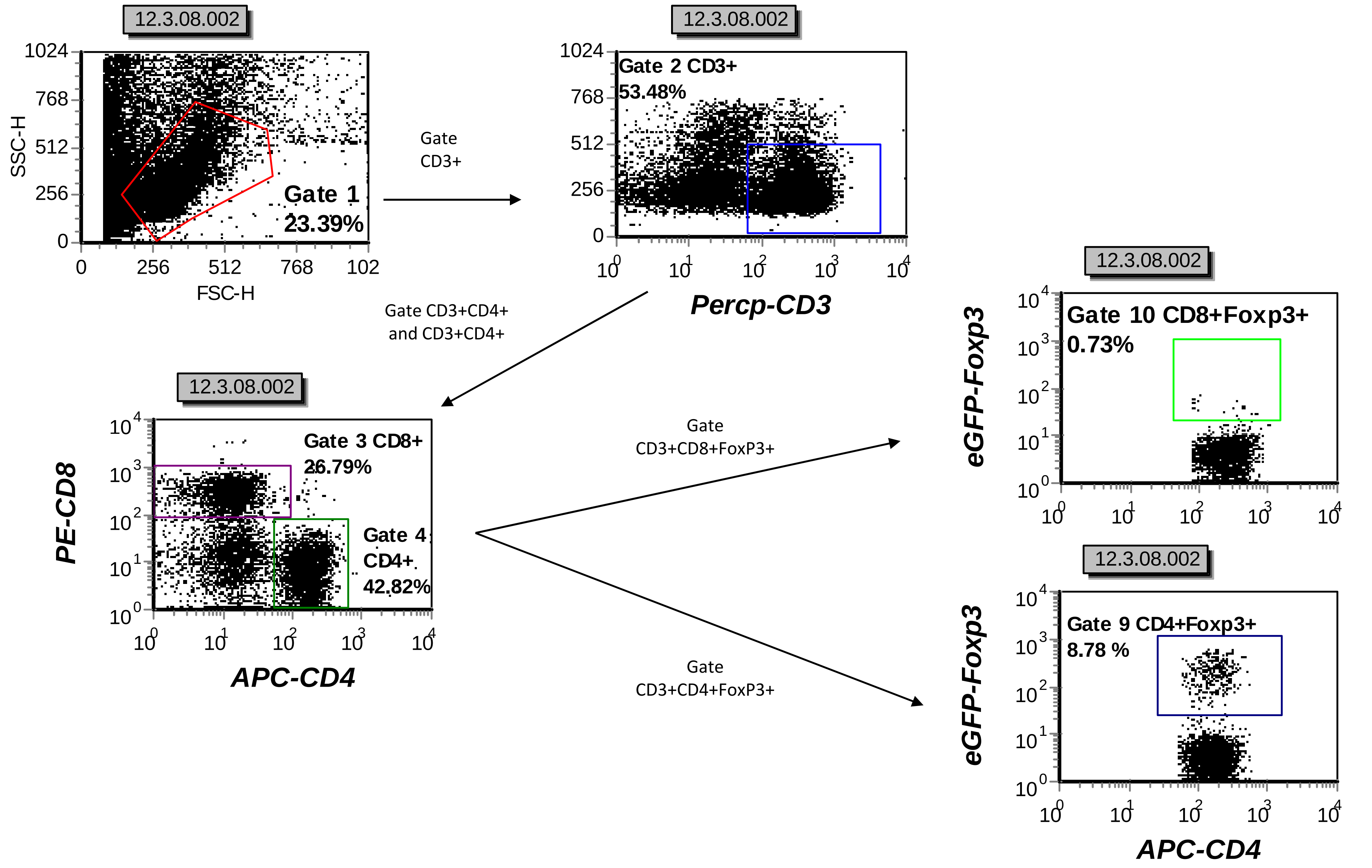

BL/6-Foxp3^{eGFP} CD4 & CD8 In Spleen (3)

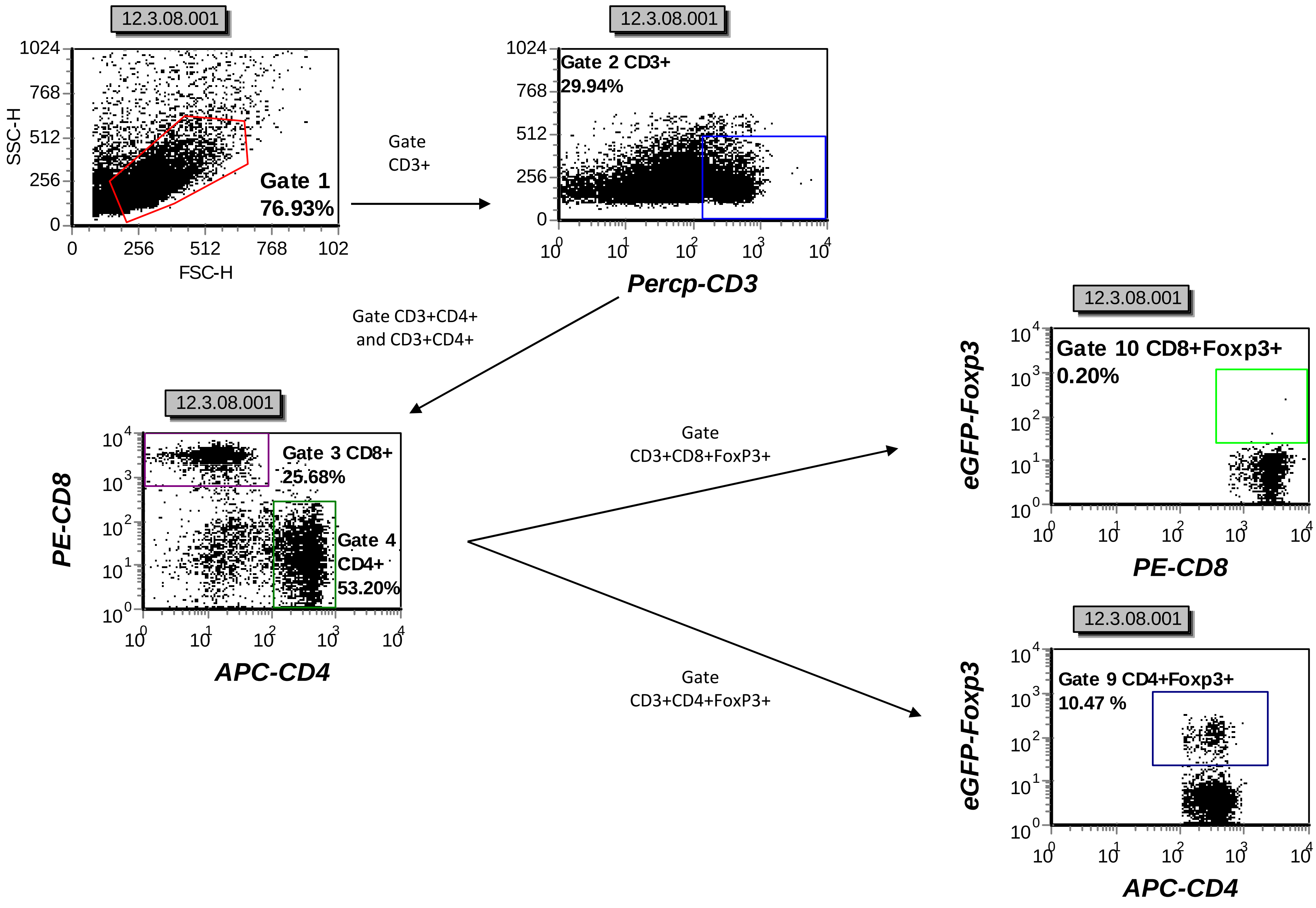

OT2-Foxp3^{eGFP} CD8 &CD4 Populations In Spleen (1)

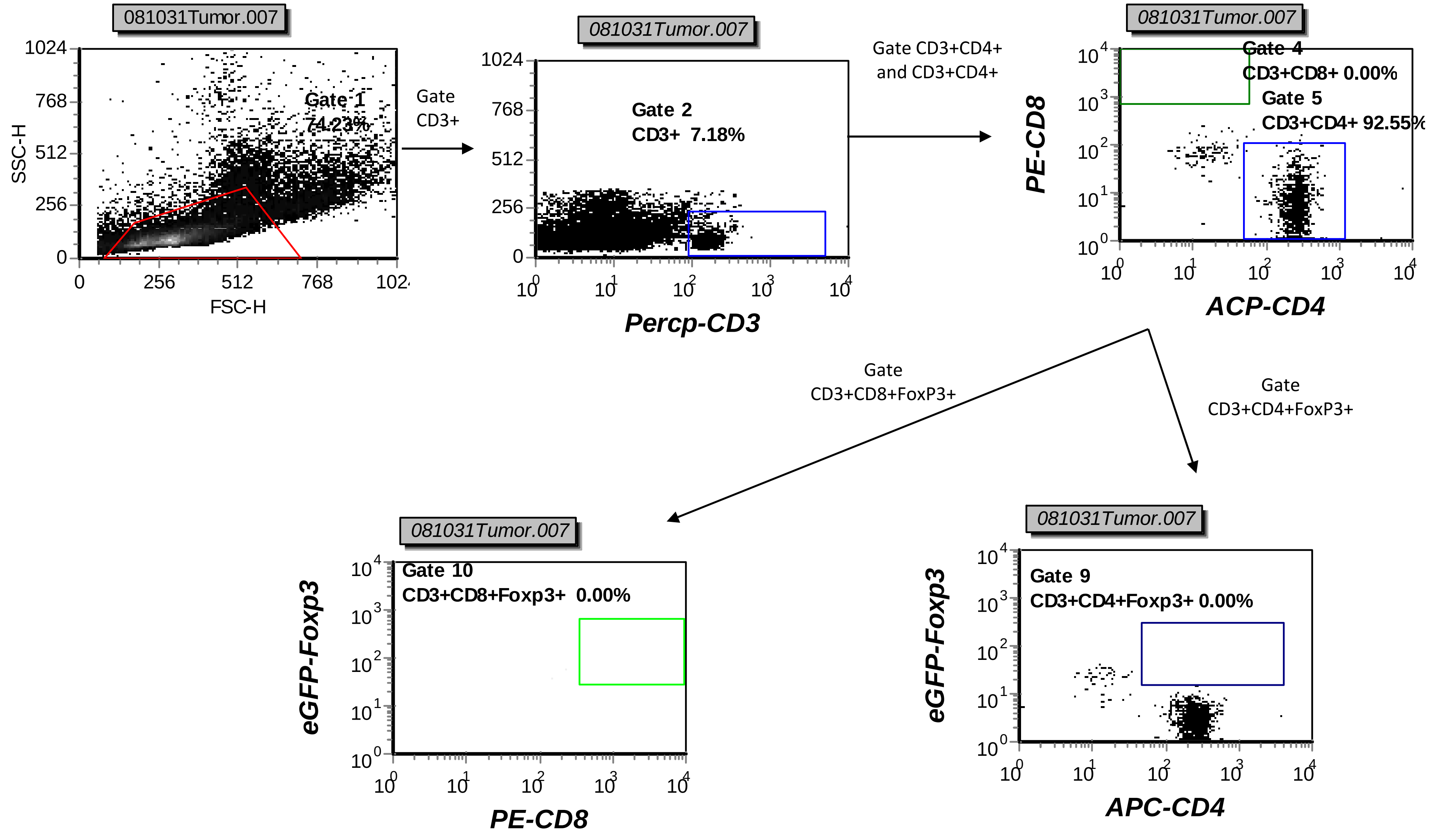

OT2-Foxp3^{eGFP} CD8 &CD4 Populations in B16-OVA Tumor (1)

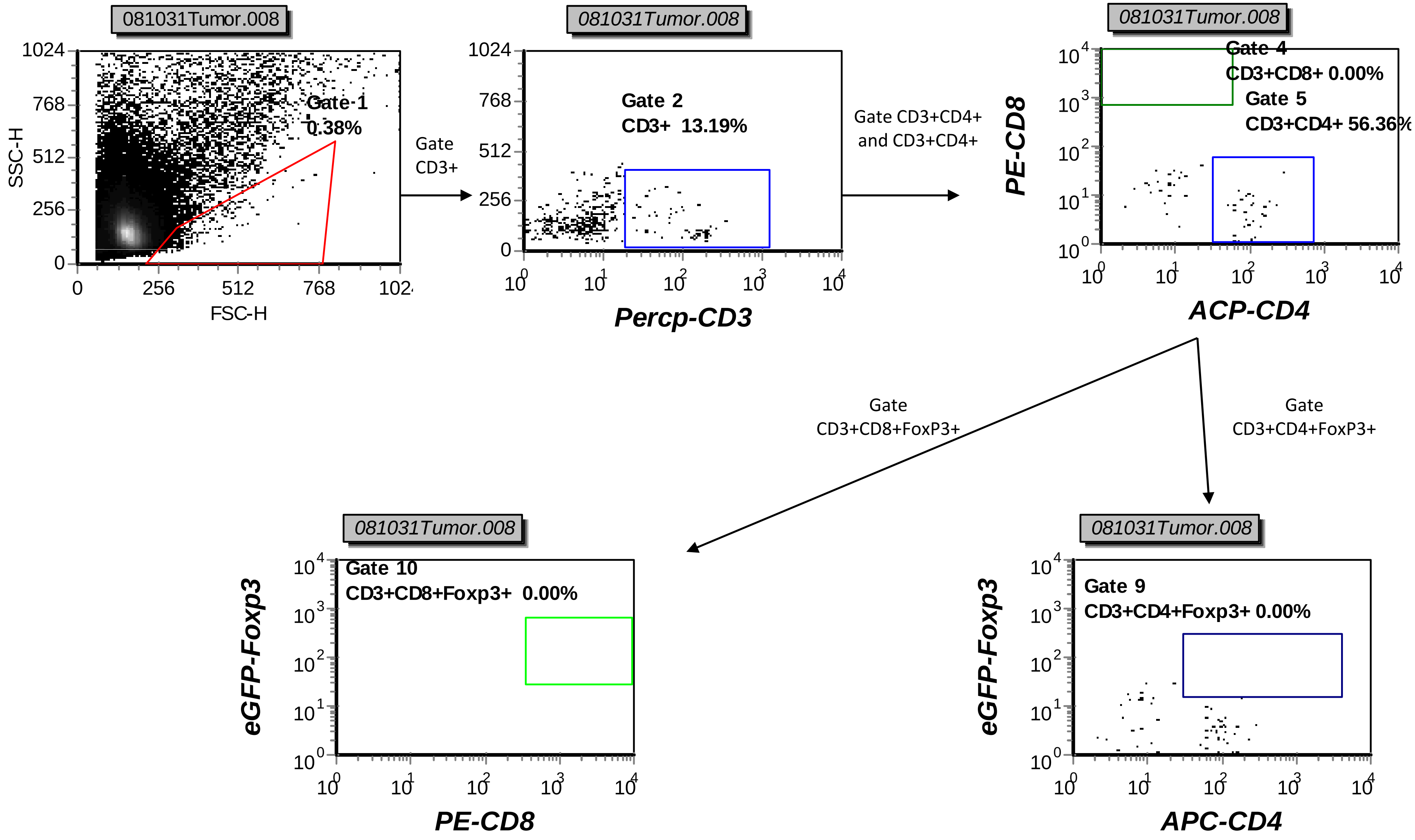

Pmel-Foxp3^{eGFP} CD8 &CD4 In Spleen (1)

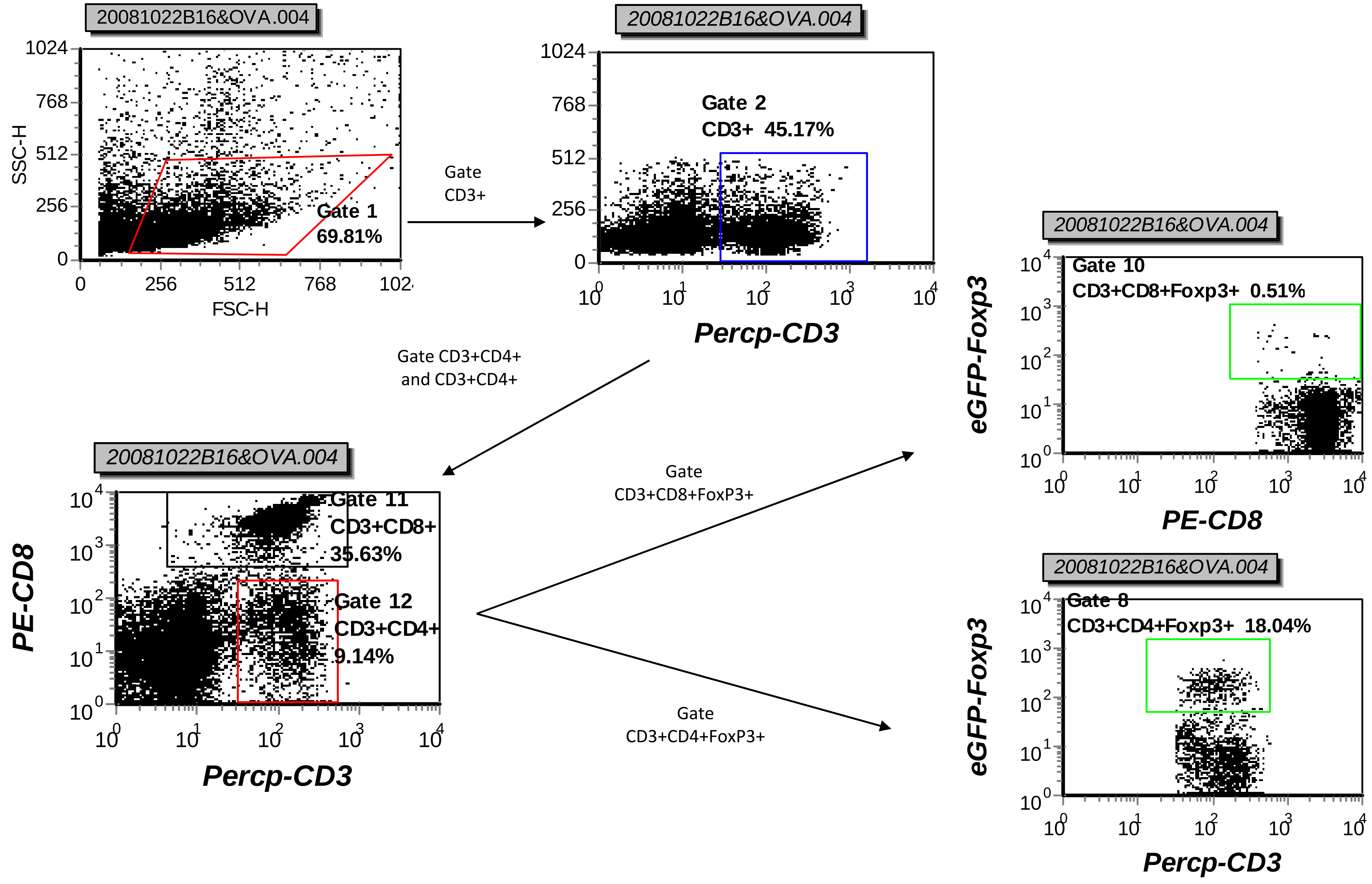

Pmel-Foxp3^{eGFP} CD4 & CD8 Populations in B16 Tumor(2)

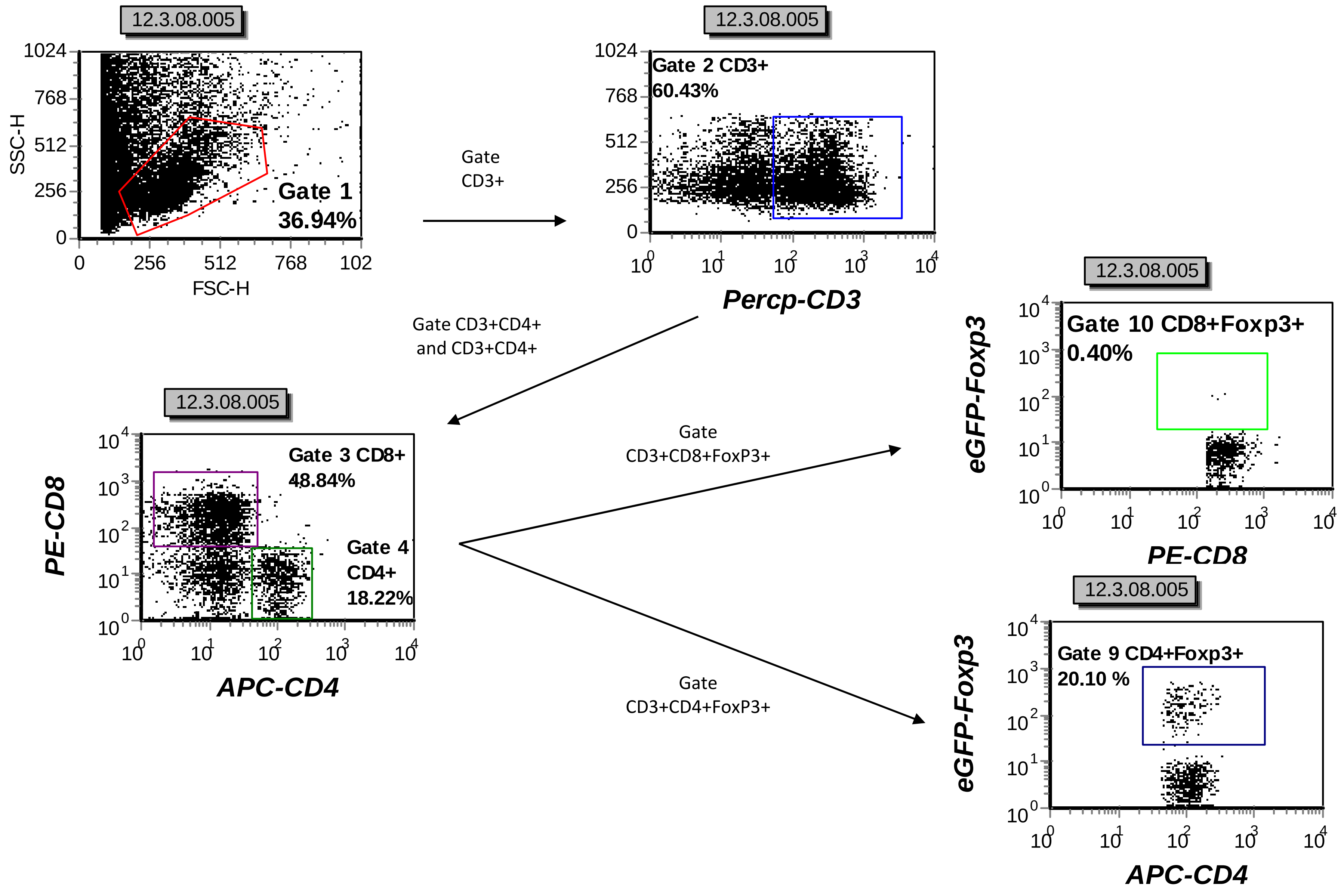

Supplement: Additional file 1 — Gating Strategies for Treg populations isolated from mice. This file shows representative examples of gating for CD8 and CD4 Foxp3EGFP cells from the spleens and tumors from different transgenic mice. These mice have been well described in previous publications where gating dot plates have been illustrated (see ref #26 which describes the generation of these mice by our coauthor T. Chatila). The numbers of infiltrating transgenic T cells varies with the size of the tumor and time after infusion. See figures 1, 2, 3, Table 2. [file 1756-8722-4-48-S1.PDF]
