# Supplementary material for: T cell receptor transgenic lymphocytes infiltrating murine tumors are not induced to express foxp3
Source: J Hematol Oncol. 2011 Nov 23;4:48. doi: 10.1186/1756-8722-4-48 (PMC3245424; doi:10.1186/1756-8722-4-48)
Supplement: Additional file 2 — Gating Strategies for Treg populations generated in vitro. This file shows representative examples of gating for CD8 and CD4 Foxp3EGFP cells generated in culture by different activated cultures. See Table 1. [file 1756-8722-4-48-S2.PDF]

BL/6-FoxP3^{GFP}

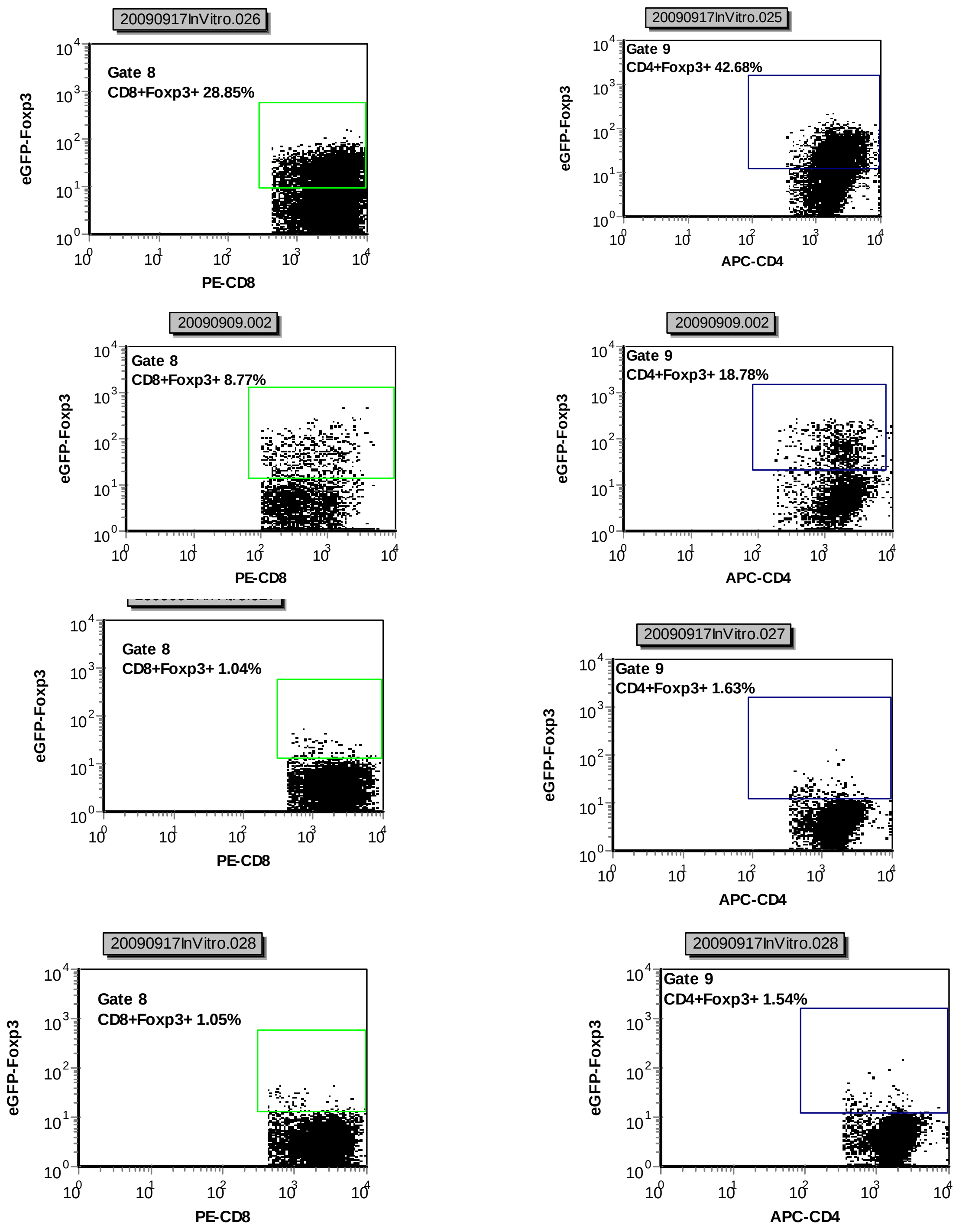

Anti-CD3/CD28/IL2/TGF-β

Anti-CD3/CD28/TGF-β

Anti-CD3/CD28/IL2

Anti-CD3/CD28

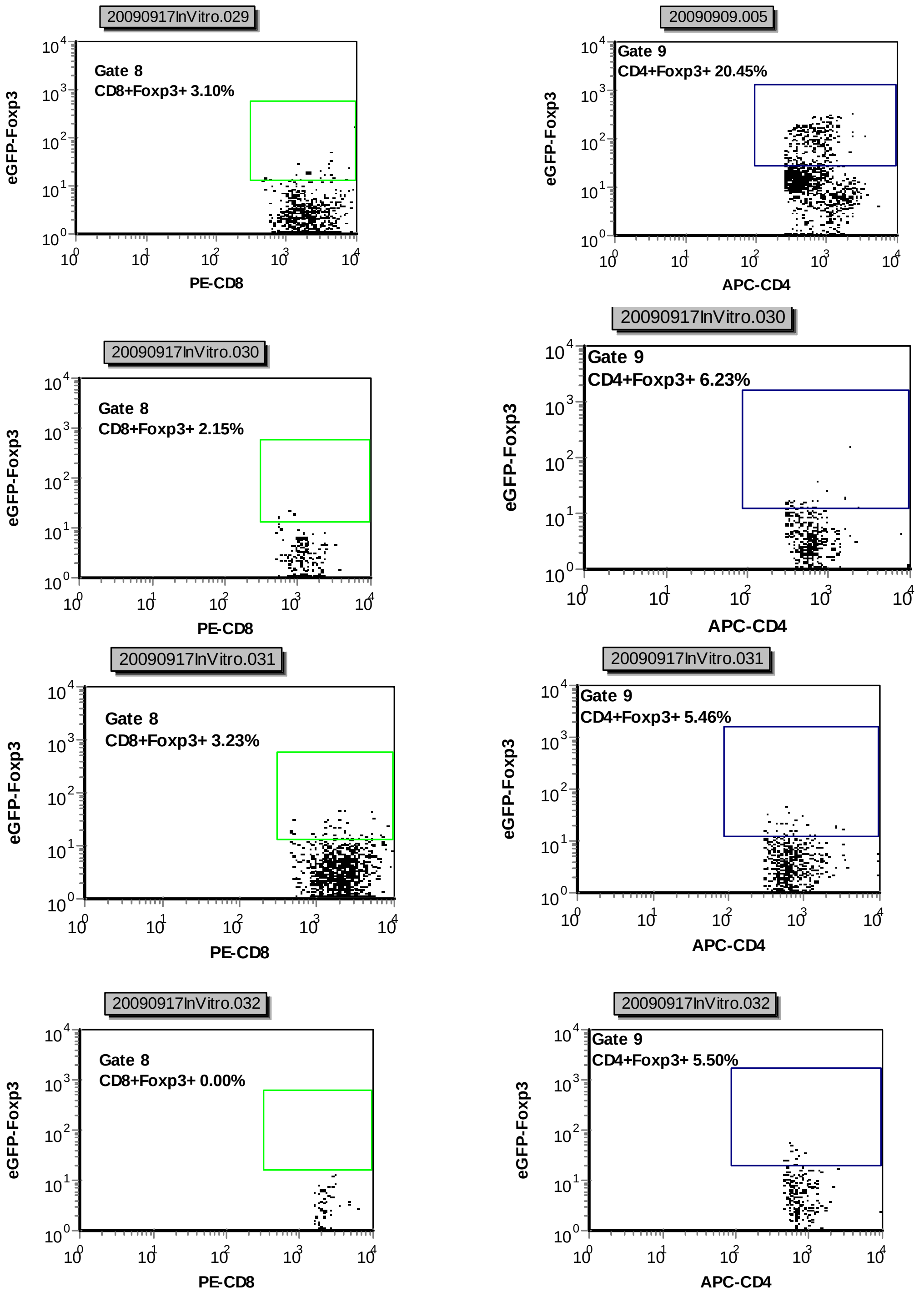

BL/6-FoxP3^{GFP}

IL2/TGF-β

TGF-β

IL2

Only Media

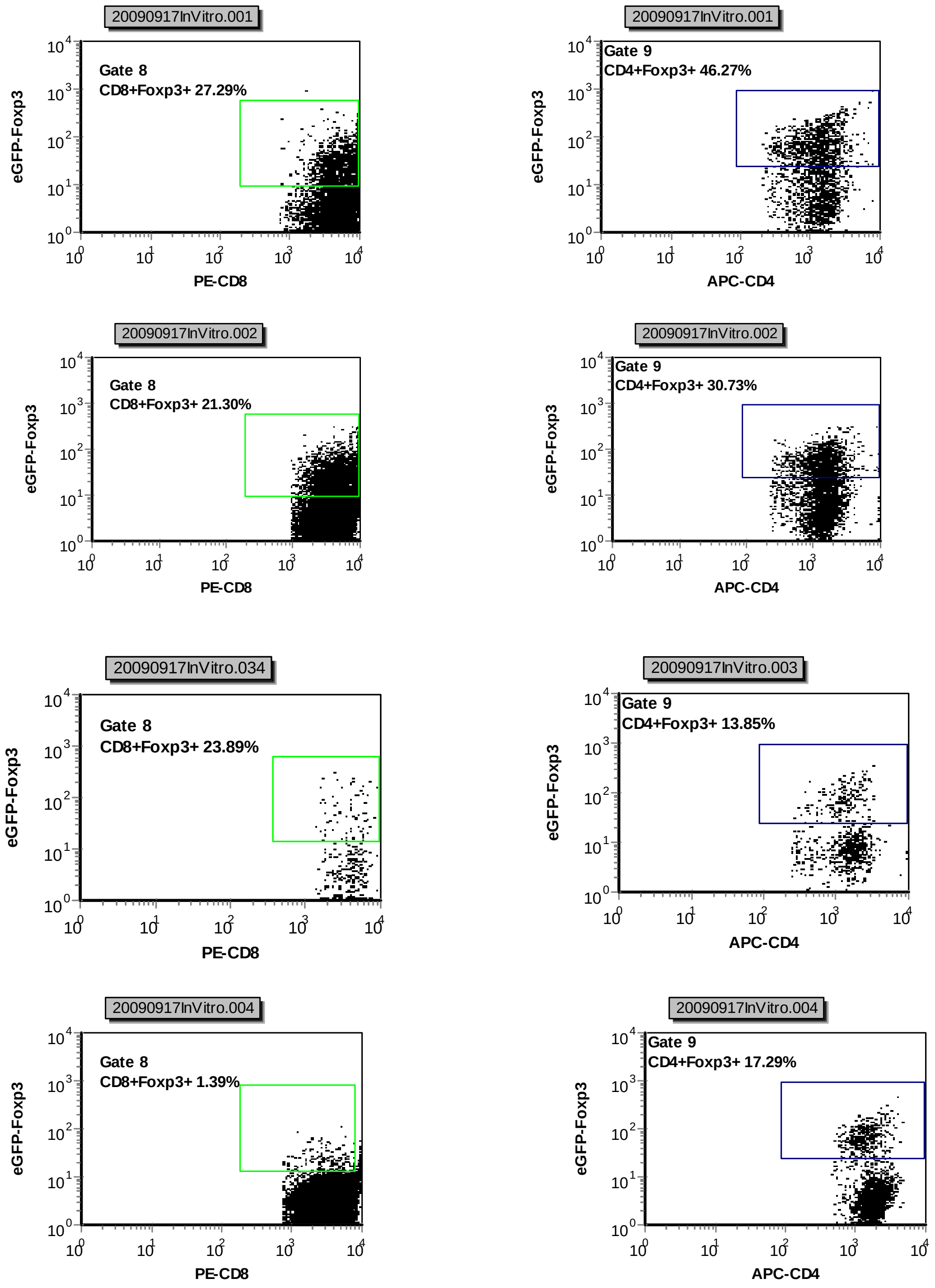

Pmel-FoxP3^{GFP}

Anti-CD3/CD28/IL2/TGF-β

Anti-CD3/CD28/TGF-β

Anti-CD3/CD28/IL2

Anti-CD3/CD28

Pmel-FoxP3^{GFP}

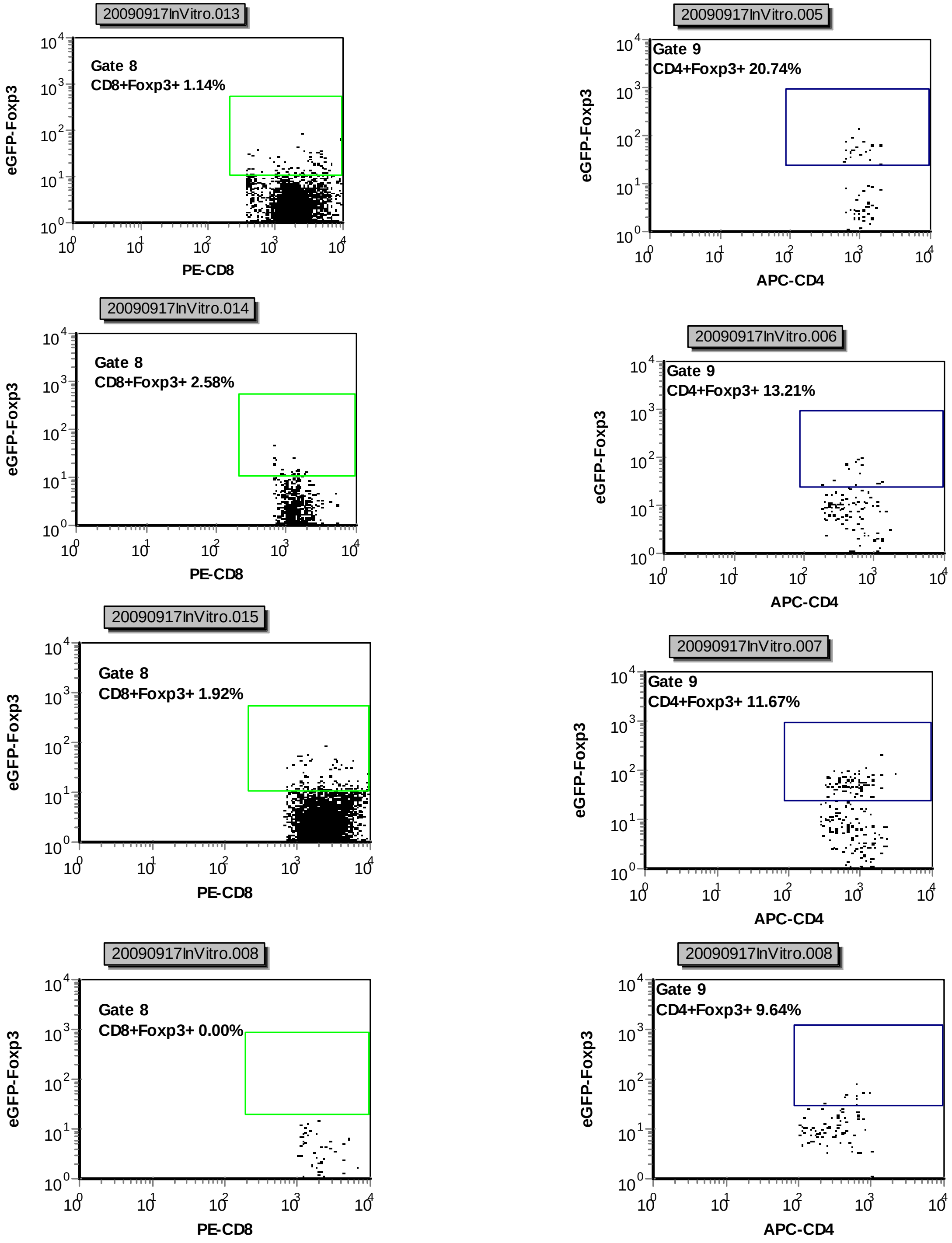

IL2/TGF-β

TGF-β

IL2

Only Media

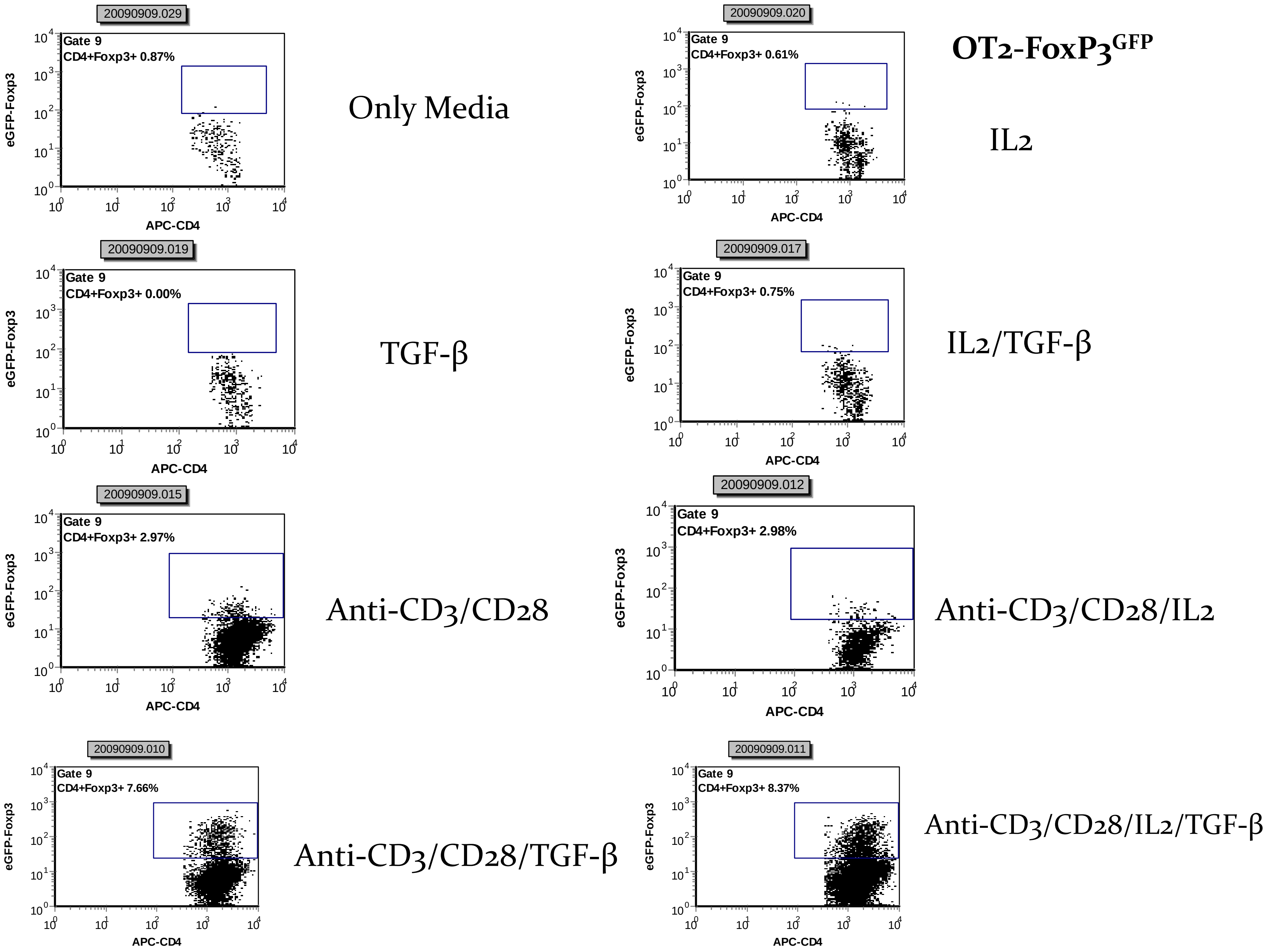
OT2-FoxP3GFP
20090909.029
Gate 9
CD4+Foxp3+ 0.87%
Only Media
20090909.020
Gate 9
CD4+Foxp3+ 0.61%
IL2
20090909.019
Gate 9
CD4+Foxp3+ 0.00%
TGF-β
20090909.017
Gate 9
CD4+Foxp3+ 0.75%
IL2/TGF-β
20090909.015
Gate 9
CD4+Foxp3+ 2.97%
Anti-CD3/CD28
20090909.012
Gate 9
CD4+Foxp3+ 2.98%
Anti-CD3/CD28/IL2
20090909.010
Gate 9
CD4+Foxp3+ 7.66%
Anti-CD3/CD28/TGF-β
20090909.011
Gate 9
CD4+Foxp3+ 8.37%
Anti-CD3/CD28/IL2/TGF-β
eGFP-Foxp3
APC-CD4
